# Furosemide-loaded Alginate Microspheres Prepared by Ionic Cross-linking Technique: Morphology and Release Characteristics

**DOI:** 10.4103/0250-474X.40336

**Published:** 2008

**Authors:** M. K. Das, P. C. Senapati

**Affiliations:** Department of Pharmaceutical Sciences, Dibrugarh University, Dibrugarh (Assam) - 786 004, India

**Keywords:** Sodium alginate, microspheres, furosemide, ionic cross-linking technique, anomalous transport mechanism, fickian diffusion controlled

## Abstract

Furosemide-loaded alginate microspheres were prepared by the ionic cross-linking technique using CaCl_2_, Al_2_(SO_4_)_3_ and BaCl_2_. The process induced the formation of microspheres with the incorporation efficiency of 65% to 93%. The effect of sodium alginate concentration, cross-linking agents and drying conditions was evaluated with respect to entrapment efficiency, particle size, surface characteristics and *in vitro* release behaviors. Infrared spectroscopic study confirmed the absence of any drug-polymer interaction. Differential scanning calorimetric analysis revealed that the drug was molecularly dispersed in the alginate microspheres matrices showing rough surface, which was confirmed by scanning electron microscopy study. The mean particle size and entrapment efficiency were found to be varied by changing various formulation parameters. The *in vitro* release profile could be altered significantly by changing various formulation parameters to give a sustained release of drug from the microspheres. The kinetic modeling of the release data indicate that furosemide release from the alginate microspheres follow anomalous transport mechanism after an initial lag period when the drug release mechanism was found to be fickian diffusion controlled.

Alginates, which are naturally occurring substances, found in brown algae have received much attention as a vehicle for controlled drug delivery^1^–^4^. Alginates can be considered as block polymers, which mainly consist of mannuronic acid (M), guluronic acid (G) and mannuronic-guluronic (MG) blocks. Dropwise addition of aqueous alginate solution to the aqueous solution containing calcium ions and/or other di and polyvalent cations cause spherical gel formation termed as alginate bead. Alginate is known to be nontoxic when taken orally and also have a protective effect on the mucous membranes of the upper gastrointestinal tract^1^. The dried alginate beads have the property of reswelling and thus they can act as controlled release system. Their reswelling property is susceptible to pH, which protects the acid-sensitive drug from gastric juice^5^.

Furosemide, a potent loop diuretic, is used in the treatment of edema of hepatic, cardiac, pulmonary and renal failures and in chronic hypertension^6^. The dose related adverse effects have been observed^6^ and the treatment with conventional tablets produce short period of maximum diuresis, which is inconvenient to the patients. But the treatment with sustained release tablets produces same diuretic effect as produced by conventional tablets eliminating the brief and intense diuresis, which is well tolarated to the patients^7^. However, such single unit sustained release tablets could be disastrous if they fail to release the drug at the desired rate and in the desired amount, or if they release the entire amount of drug so as to cause dose dumping. The multiunit microparticulate oral drug delivery systems can be distributed widely throughout the gastrointestinal tract providing a possibility of achieving a longer lasting and reliable release of drug at desired rate. Unwanted intestinal retention of the polymeric material and local irritation which may occur with non-disintegrating polymeric matrix tablets, can also be avoided^8^.

As previously reported, the furosemide microspheres prepared by emulsion-solvent evaporation method utilize larger volume of organic solvents^9^, which are costly and hazardous because of the possibility of explosion, toxicity and air pollution. The water-based ionic cross-linking technique can provide characteristic advantages over conventional microspheres preparation methods. As previously reported, this technique can be used successfully to prepare alginate microspheres containing acetaminophen^10^ and nimesulide^11^. In the present study, the same procedure was applied to prepare furosemide-loaded alginate microspheres. The effects of factors, such as sodium alginate concentrations, cross-linking agents, drying conditions, on morphology of and drug release from microspheres were studied. Drug-polymer interactions in the solid state were studied by infrared spectrophotometry (IR) and differential scanning calorimetric analysis (DSC) and the surface characteristics were evaluated by scanning electron microscopy (SEM).

## MATERIALS AND METHODS

Furosemide was received as a gift sample from Cipla Ltd., Mumbai. Sodium alginate was procured from Loba Chemie, Mumbai. Calcium chloride (fused), barium chloride and aluminium sulphate were purchased from Ranbaxy Laboratories Ltd., New Delhi. All other chemicals and solvents were of analytical grade satisfying pharmacopoeial specifications.

### Formulation of alginate microspheres:

The microspheres were prepared by ionic cross-linking technique using the formulations as shown in Table 1. The alginate solutions comprising 2.5% to 4% w/v sodium alginate were prepared by initially dissolving the polymer in deionized water using gentle heat, being stirred magnetically. On complete solution, an accurately weight quantity of furosemide was added to each solution to afford homogeneous dispersions. The dispersions were sonicated for 30 min to remove any air bubbles that may have been formed during the stirring process. The sodium alginate-drug dispersions (25 ml) were added drop wise via a 20-gauge hypodermic needle fitted with a 10 ml syringe into 50 ml of 5% w/v of cross-linking agents, being stirred at 200 rpm for 10 min. The cross-linking agents were used CaCl_2_, Al_2_(SO_4_)_3_ and BaCl_2_. The droplets from the dispersions instantaneously gelled into discrete furosemide-alginate matrices upon contact with the solution of cross-linking agents. The formed alginate microspheres were further allowed to stir in the solution of cross-linking agents for an additional 1 h. On expiration of this period the solution of cross-linking agents was decanted and the microspheres were washed with 3 × 50 ml volumes of deionized water. The microspheres were thereafter dried at 80° for 2 h in a hot-air oven. Similarly, air-dried alginate microspheres (formulation code C1, E1 and F1) were prepared in the same way as the formulations C, E and F, respectively, by drying in air at room temperature.

**TABLE 1:** FORMULATION OF ALGINATE MICROSPHERES

F.N.Code	Furosemide (%w/v)	Sodium alginate (%w/v)	Cross-linking agents (5%w/v)
A	2.5	2.5	CaCl_2_
B	2.5	3.0	CaCl_2_
C	2.5	3.5	CaCl_2_
D	2.5	4.0	CaCl_2_
E	2.5	3.5	Al_2_(SO_4_)_3_
F	2.5	3.5	BaCl_2_

### Morphology and size distribution:

The shape and surface morphology of the alginate microspheres were investigated using Jeol, JSM-6360, scanning electron microscope at 15 kV. Prior to examination samples were gold coated under vacuum (Fine coat, Ion sputter, JFC-1100) to render them electrically conductive. The samples include various alginate microspheres prepared using different cross-linking agents before release study. The alginate microspheres were not subjected to SEM studies after release study because they converted to gel type of matrix when dissolution was overed.

Size and size distribution of the alginate microspheres were measured by sieve analysis. The alginate microspheres were separated into different size fractions (% weight fraction) by sieving for 5 min using standard sieves having nominal mesh apertures of 1.4 mm, 1.2 mm, 1.0 mm, 0.85 mm and 0.71 mm (sieve no. 12, 14, 16, 18 and 22, respectively). The particle size distributions of the microspheres were determined and the mean particle size of microspheres were calculated using the following formula^12^: $\text{Mean particle size = }\sum \frac{\text{mean particle size of the fraction }\times \text{ weight fraction}}{\text{weight fraction}}$

### Determination of drug incorporation efficiency:

Thirty milligrams of drug-loaded alginate microspheres from each batch was placed in 100 ml conical flask containing 50 ml of phosphate buffer of pH 7.4. The microspheres were magnetically stirred to promote swelling and break up of the cross-linked structure. This afforded liberation and subsequent dissolution of furosemide. The solution was filtered through a 0.45 μm membrane filter. Then the drug was quantified at 276.5 nm spectrophotometrically after appropriate dilution with phosphate buffer of pH 7.4. The incorporation efficiency was determined by the following empirical relationship: Drug incorporation efficiency (%) = (AQ/TQ) × 100, where AQ is the actual quantity of drug present in the microspheres and TQ is the 100% theoretical quantity of drug present in the microspheres (i.e., actual initial loading dose).

### Infrared spectroscopy:

The drug-polymer interactions were studied by infrared spectroscopy. The IR spectra were recorded between 500 to 4000 cm^−1^ for pure furosemide, blank alginate microspheres and furosemide-loaded alginate microspheres from KBr pellet using Perkin Elmer-883 IR spectrophotometer.

### Differential scanning calorimetry:

The DSC thermograms were recorded on a Universal V 2.5 H differential scanning calorimeter. The DSC studies on the samples were performed by heating samples at a heating rate of 10°/min over a temperature range of 50-300° in closed aluminium pans. The samples included pure furosemide and furosemide-loaded alginate microspheres.

### *In vitro* dissolution testing:

The dissolution studies were performed in a fully calibrated six station dissolution test apparatus (37 ± 0.5°, 50 rpm) using the USP rotating basket method in phosphate buffer media (pH 7.4, 500 ml). A quantity of alginate beads equivalent to 100 mg furosemide for each formulation was employed in all dissolution studies. The samples of 5 ml each were withdrawn at predetermined time interval and were replenished immediately with the same volume of fresh prewarmed phosphate buffer maintaining sink condition throughout the experiment. The aliquots, following suitable dilution, were analyzed spectrophotometrically at 276.5 nm. The concentrations of furosemide in the test samples were calculated using a regression equation (Absorbance = 0.007 + 0.0709 × concentration, R^2^ = 0.999) of the calibration curve in phosphate buffer of pH 7.4 and corrected for sampling effect using the formula reported by Hayton and Chen^13^.

### Kinetic modeling:

In order to investigate the release mechanism, the release data (≤60%) were fitted to the following power law expression^14^: M_t_/M_∞_ = Kt^*n*^ …(1), where M_t_ and M_∞_ are the amounts of drug released at time t and the overall amount released, respectively, K is the release rate constant and *n* is the release exponent indicative of release mechanism. The release data were further analyzed using the modified form of the power law expression^15^ to accommodate the lag time (t_L_) in the beginning of the drug release from the alginate beads: M_t_/M_∞_ = K(t − t_L_)^*n*^ …(2). Fitness of the data into various kinetic models was assessed by determining the correlation coefficient. The value of *n* was calculated from the slope of the plot of log (M_t_/M_∞_) *vs*. log (t) and log (M_t_/M_∞_) *vs*. log (t−t_L_) for interpretation of release mechanism^16^ (Table 3).

**TABLE 3:** FUROSEMIDE RELEASE KINETIC DATA

F.N.Code	M_t_/M_∞_ = Kt^n^ model		M_t_/M_∞_ = K(t−t_L_)^n^ model		Drug release mechanism
			
	R^2^	*n*	R^2^	*n*
A	0.9308	1.997	0.9290	0.9980	0.5 < *n* > 1.0 Anomalous transport, super case II transport
B	0.9910	1.553	0.9630	0.8360	0.5 < *n* > 1.0 Anomalous transport, super case II transport
C	0.9350	0.9360	0.9840	0.9650	0.5 < *n* < 1.0 Anomalous transport
D	0.9360	0.9160	0.9820	0.9350	0.5 < *n* < 1.0 Anomalous transport
E	0.8970	0.3050	0.9870	1.06	0.5 > *n* ≥ 1.0 Fickian diffusion, case II/super case II transport
F	0.9600	1.17	0.9820	1.40	*n* > 1.0 Super case II transport
C1	0.8490	0.542	0.9870	1.383	0.5 ≤ *n* > 1.0 Fickian diffusion, super case II transport
E1	0.8310	0.20	0.9920	1.110	0.5 > *n* > 1.0 Fickian diffusion, super case II transport
F1	0.8480	0.3440	0.9690	1.179	0.5 > *n* > 1.0 Fickian diffusion, super case II transport

R^2^ is the correlation coefficient and *n* is the release exponent

## RESULTS AND DISCUSSION

**Fig. 1:**
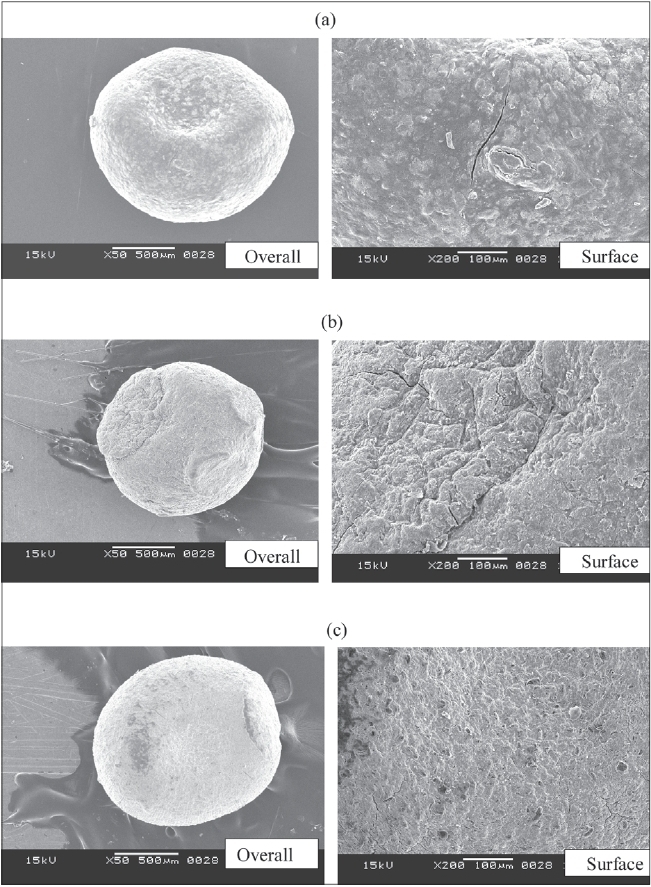
SEM of furosemide-loaded alginate microspheres. SEM of furosemide-loaded alginate microspheres, (a) calcium alginate microsphere, (b) aluminum alginate microsphere and (c) barium alginate microsphere.

**Fig. 2:**
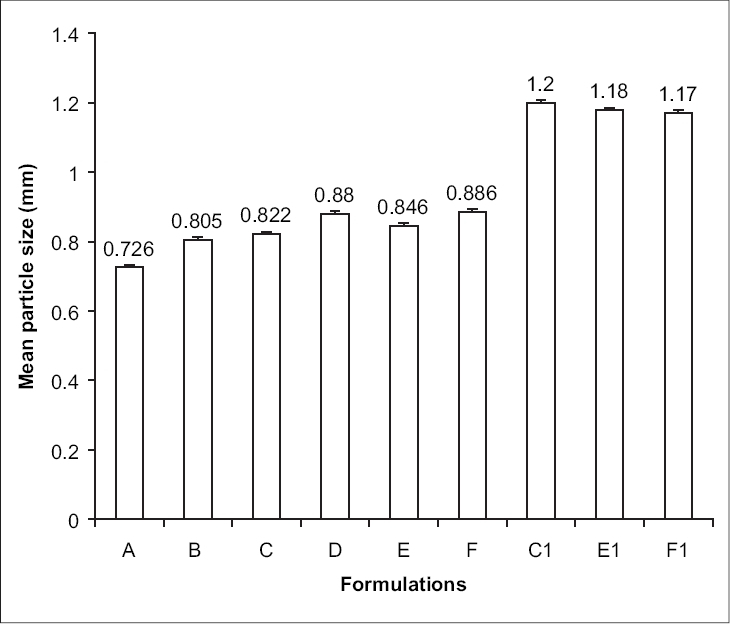
Effect of polymer concentrations, cross linking agents and drying conditions on the microspheres size.

Morphology of the various formulations of alginate microspheres prepared was found to be discrete and spherical in shape. The SEM photomicrographs of the dried alginate microspheres are shown in fig. 1. The surface of the alginate microspheres was rough due to higher concentration of drug uniformly dispersed at the molecular level in the alginate matrices. The mean particle size of the various formulation of alginate microspheres were between 0.726 ± 0.007 to 1.2 ± 0.008 mm. It was found that the particle size distribution of each formulation was within a narrow range but the mean particle size was different among the formulations (fig. 2.) The results indicated the proportional increase in the mean particle size of the microspheres with increasing amount of sodium alginate in the formulations A, B, C and D. This could be attributed to an increase in the relative viscosity at higher concentration of sodium alginate and formation of large droplets during addition of the polymer solution to the cross-linking agents. Air dried microspheres were of larger size than those oven dried due to incomplete dehydration as a result of air drying process. The results also indicated no significant variation in particle size of the microspheres prepared using different cross-linking agents.

The incorporation efficiency increased progressively with increasing sodium alginate concentration (Table 2). Increase in the alginate concentration resulted in the formation of larger microspheres entrapping greater amounts of the drug. This may be attributed to the greater availability of active calcium-binding sites in the polymeric chains and, consequently, the greater degree of cross-linking as the quantity of sodium alginate increased^17^. The incorporation efficiencies were generally higher for the formulations cross-linked with Al_2_(SO_4_)_3_ and BaCl_2_ as compared to the beads cross-linked with CaCl_2_. The results tabulated in Table 2 indicate that the incorporation efficiencies were more than 90% for both oven-dried and air-dried alginate beads cross-linked with Al_2_(SO_4_)_3_ and BaCl_2_. This may be attributed to the formation of nonporous alginate beads due to increase in the apparent cross-linking density in presence of Al^3+^ and Ba^2+^ which prevent the diffusion of the drug out of the beads at the time of curing. The low incorporation efficiency of alginate beads cross-linked with Ca^2+^ could be attributed to the formation of porous beads ensuring the diffusion of the drug out of the beads at the time of curing.

**TABLE 2:** INCORPORATION EFFICIENCY OF ALGINATE MICROSPHERES

F.N. Code	Entrapment capacity (%w/w) (mean* ± SD)
A	65.18 ± 5.4
B	66.10 ± 2.26
C	72.01 ± 0.28
D	71.25 ± 1.93
E	91.00 ± 1.00
F	91.78 ± 0.233
C1	74.25 ± 0.84
E1	91.63 ± 0.021
F1	93.00 ± 1.41

* *n* = 3

**Fig. 3:**
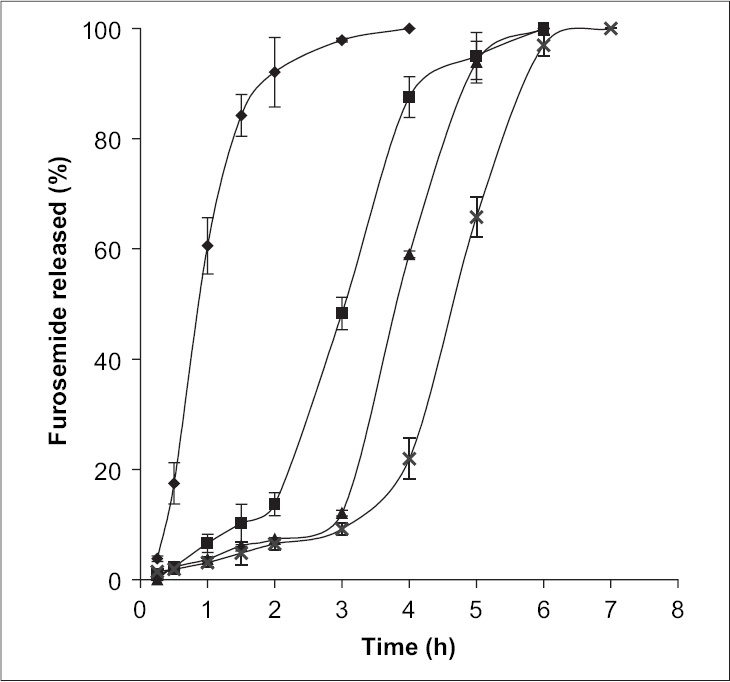
Effect of sodium alginate concentration on release characteristics of furosemide. Effect of sodium alginate concentration on release characteristics of Furosemide in phosphate buffer pH 7.4. 2.5% w/v (◆), 3% w/v (■), 3.5% w/v (▲) and 4% w/v ( × ). Bars indicate ± SD (*n* = 3).

To study the effect of sodium alginate concentration on furosemide release, the sodium alginate was used at four different concentrations: 2.5 (A), 3.0 (B), 3.5 (C) and 4.0 (D) % w/v. The release profiles for these formulations are shown in fig. 3. The results indicated the more sustained effect with increase in the concentration of sodium alginate. It is observed from the fig. 3 that the steady state release was achieved after an initial lag time and it was directly proportional to the concentration of sodium alginate. This type of release behavior agreed with the pulsatile release pattern. The pulsatile or pulsed drug release is defined as the rapid release of certain amount of drug within a short time period immediately after a lag time^18^. The pulsed release pattern could be controlled by the alginate gel disintegration in phosphate buffer. The alginate disintegration was monitored by the exchange of Ca^2+^ with Na^+^ in the dissolution medium. The first phase (negligible release portion of the release graph, lag time) might be for the negligible dissociation of alginate beads in phosphate buffer and the drug release mainly based on drug diffusion through the small pores and cracks. The second phase exhibited a burst-like release pattern, which was accompanied by alginate disintegration. The sodium alginate concentration in the formulation greatly influenced the steady state release of furosemide from the alginate beads. The principle of gelation or cross-linking of sodium alginate with calcium chloride is based on the formation of tight junction between the guluronic acid residues^4^. The number of the apparent cross-linking points formed within the calcium alginate gel beads increased with increasing alginate concentration in the formulation. This increase in the apparent cross-linking density delayed the alginate gel disintegration in phosphate buffer due to the retardation of Ca^2+^ exchange with Na^+^ and eventually increasing lag time. Increased alginate gel density per unit volume was also thought to affect the decreased pore size within the gels, and thus furosemide release becomes slow.

**Fig. 4:**
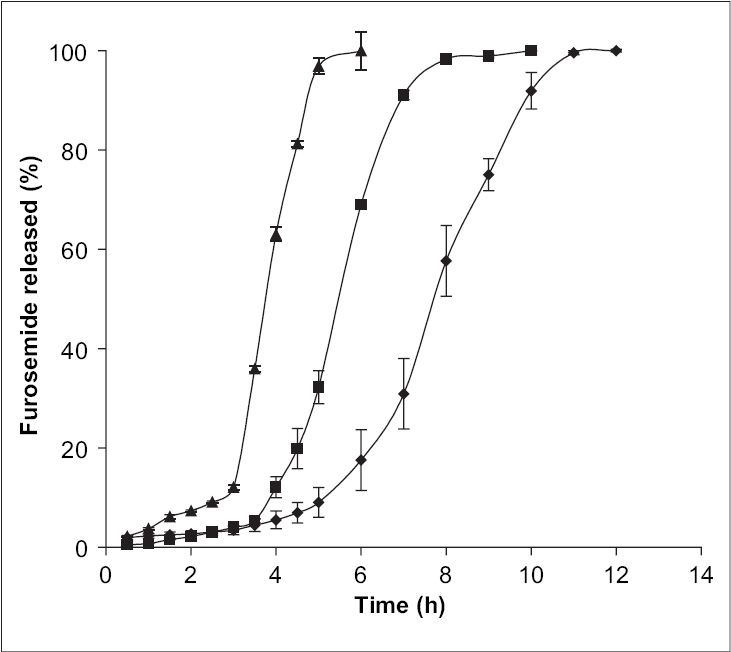
Effect of various cross linking agents on release characteristics of furosemide from alginate beads. Effect of various cross-linking agents at 5% w/v level on release characteristics of Furosemide from alginate beads in phosphate buffer pH 7.4. Al_2_ (SO_4_)_3_ (◆), BaCl_2_ (■) and CaCl_2_ (▲). Bars indicate ± SD (*n* = 3).

The release behaviors of alginate microspheres, produced by ionic gelation with different cross-linking agents depend upon the valency and size of the cations of the respective cross-linking agent^19^. To investigate this aspect, the sodium alginate (3.5% w/v) beads were prepared via cross-linking in 5% w/v solution of CaCl_2_ (C), Al_2_(SO_4_)_3_ (E) and BaCl_2_ (F), respectively. Their release profiles in phosphate buffer of pH 7.4 have been well depicted in the fig. 4. The pulsatile release pattern was observed in all cases. The steady state release was achieved after 2.45 h for Ca^2+^-alginate microspheres, 3.5 h for Ba^2+^-alginate microspheres and 5.0 h for Al^3+^-alginate microspheres. The results obtained can be explained on the basis of the extent of cross-linking in the microspheres. Ca^2+^ and Ba^2+^, being divalent, form two-dimensional bonding structure with sodium alginate inside the alginate matrices. But, Ba^2+^ has largest size (1.74 A°) as compared to the other two cations (1.14 A° for Ca^2+^ and 0.68 A° for Al^3+^), it is expected to form strong alginate microspheres with smaller voids and low water uptake^19^. Therefore, the exchange of larger Ba^2+^ in the microspheres with Na^+^ of dissolution medium (phosphate buffer, pH 7.4) and also their removal in the form of insoluble barium phosphate was hindered, thus resulting in delayed swelling of the microspheres and slow release. In case of Ca^2+^-alginate microspheres, the smaller size of Ca^2+^ as compared to Ba^2+^ ensure rapid removal of Ca^2+^ as calcium phosphate from the microspheres due to ion-exchange process with Na^+^ of phosphate buffer medium and thus leading to greater water uptake and rapid release. In case of Al^3+^-alginate microspheres, the delay was due to the ability of Al^3+^ to form three-dimensional bonding structure with the sodium alginate inside the microspheres. This three-dimensional bonding results in extended cross-linking through the whole microsphere producing hard alginate microspheres with low water uptake and thus leading to slow removal of Al^3+^ due to ion-exchange with Na^+^ in the phosphate buffer. As a result, the swelling of the beads are delayed leading to slow disintegration.

**Fig. 5:**
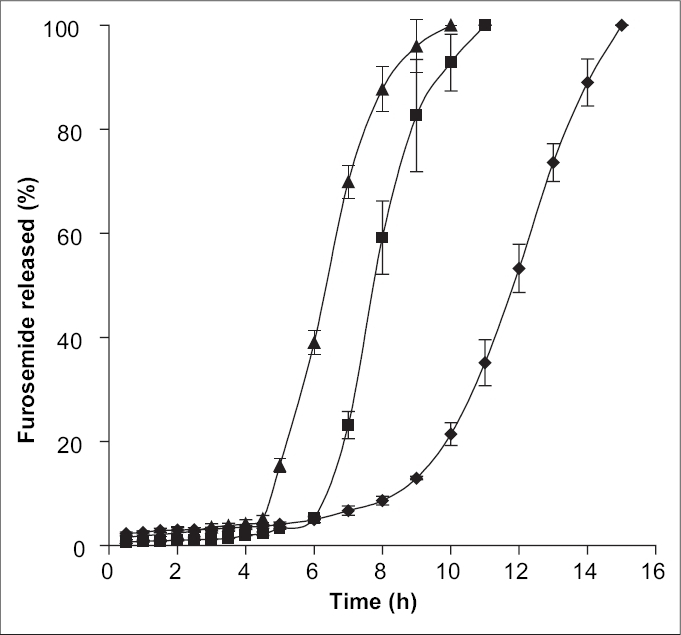
*In vitro* release profiles of furosemide from air-dried alginate beads. Release profiles of furosemide from air-dried alginate beads prepared with Al_2_ (SO_4_)_3_ (◆), BaCl_2_ (■ and CaCl_2_ (▲) at 5% w/v level. Bars indicate ± SD (*n* = 3).

The influence of the degree of dehydration on the release of furosemide was investigated by drying alginate microspheres (formulation C, E and F) at 80° for 2 h in a hot air oven, whilst the duplicate batches (formulations C1, E1 and F1) were dried in air at room temperature. The release of furosemide from oven-dried alginate microspheres was observed to take place at a faster rate as compared to the air-dried alginate microspheres (figs. 4 and 5), because the steady state release was achieved after 5 h for air-dried Ca^2+^-alginate microspheres, where as the value was 6 h and 8.3 h for air-dried Ba^2+^-alginate and Al^3+^-alginate microspheres, respectively. The alginate microspheres that had been heated at 80° for 2 h appeared to have become fully dehydrated. The complete dehydration of alginate microspheres may develop a small degree of surface cracking which can facilitate the surface erosion of the beads upon rehydration^20^ and consequently, furosemide release was more rapid from the oven-dried microspheres as compared to the air-dried microspheres. Air-drying produced partially hydrated alginate microspheres due to incomplete dehydration and the particle size of the microspheres was larger than the oven-dried microspheres. The slow release from the larger air-dried microspheres may be due to the reduction in dissolution surface area. Also, the incomplete dehydration may significantly reduce the pore size of the alginate microspheres and may prevent the surface cracking.

The *in vitro* dissolution data were analyzed by different kinetic models in order to find out the *n* value, which describe the drug release mechanism. The values of *n* and the coefficient of correlation (R^2^) obtained for the respective model are listed in the Table 3. The best fit with the highest correlation coefficient was shown in modified power law expression (Eqn. 2). The values of *n* for the release of furosemide from the alginate microspheres range between 0.8360 to 1.383 (from Eqn. 2), indicating that the drug release from the microspheres followed the anomalous transport and super case-II transport mechanism controlled by swelling and relaxation of the polymer chains. For the formulation E, C1, E1 and F1, the *n* values from power law expression (Eqn. 1) range between 0.20 to 0.54, indicating the mechanism of the initial drug release to be diffusion controlled during the lag period, when alginate dissociation was almost negligible in the dissolution medium.

**Fig. 6:**
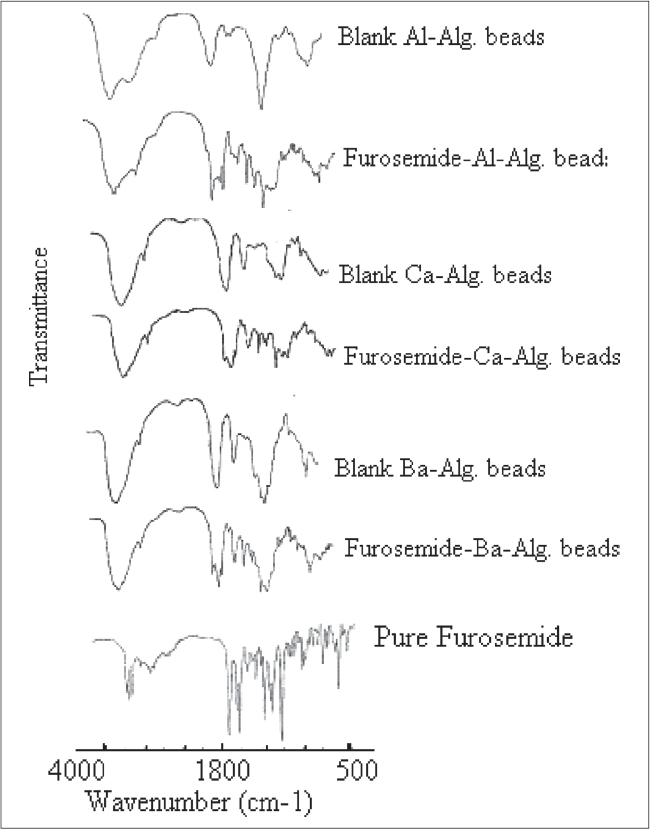
IR spectra of blank and furosemide-loaded alginate beads and pure furosemide. IR spectra of blank calcium alginate beads, furosemide-loaded calcium alginate microspheres, blank aluminium alginate microspheres, furosemide-loaded aluminium alginate microspheres, blank barium alginate microspheres, furosemide-loaded barium alginate microspheres and pure furosemide.

The stability of furosemide in the alginate microspheres was investigated by infrared spectroscopy study (IR). The study of IR spectra of furosemide (fig. 6) demonstrated that the characteristics absorption bands for N-H stretching vibration of secondary amine, C = O stretching vibration and S = O stretching vibration of sulphonamide group and C-Cl stretching vibration appeared at 3398, 1675, 1593, 1325 and 578 cm^−1^, respectively. The almost identical absorption bands were obtained from furosemide-loaded alginate microspheres, but with lower intensity as shown in fig. 6. The above observed absorption bands were similar to the reported values^7^. Thus, the IR study indicates the stable nature of furosemide in the freshly prepared alginate microspheres.

**Fig. 7:**
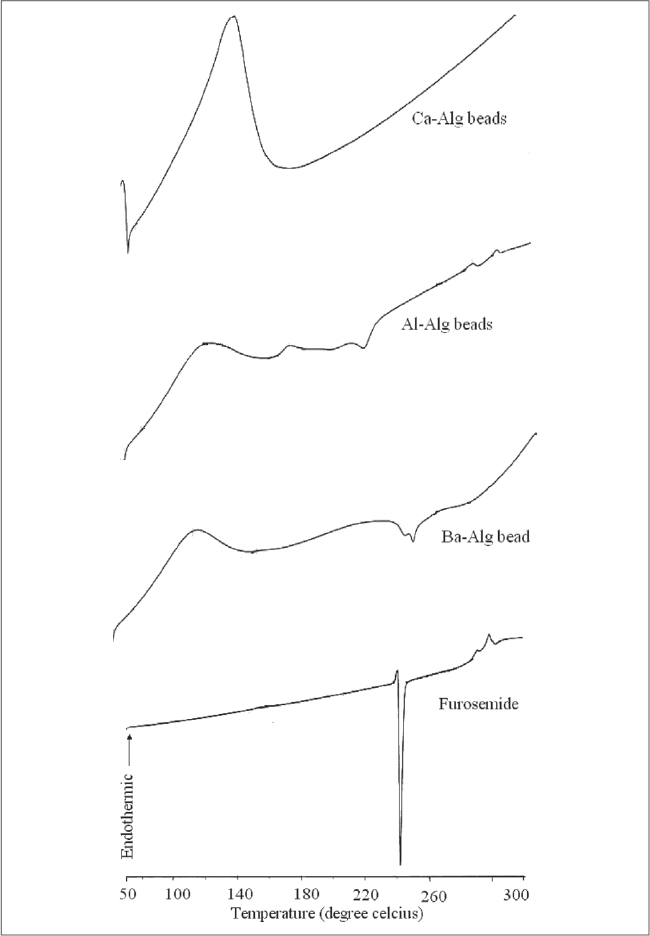
DSC thermograms of pure drug and drug-loaded alginate microspheres. DSC thermograms of pure drug and drug-loaded alginate microspheres prepared using different cross-linking agents.

The DSC thermograms of pure drug and drug-loaded alginate microspheres prepared with different cross-linking agents are shown in fig. 7.. Furosemide exhibited a sharp endothermic peak at 220.8°C corresponding to its melting point. The peak of the drug did not appear in the thermogram of any type of the prepared microspheres containing the drug. It may indicate that the drug was uniformly dispersed at the molecular level in the microspheres as observed by SEM analysis (fig. 1).

It can be concluded from the above investigation that the proper selection of formulation conditions are very important to achieve high encapsulation efficiency and to control the release of furosemide from alginate microspheres. The pulsatile release pattern was observed from all the formulations investigated. The alginate microspheres swelled and eventually disintegrated in phosphate buffer of pH 7.4. Consequently, 100% of furosemide was released in the dissolution medium. Therefore, more formulation studies are needed to design the best controlled release formulation.
